# Mechanisms of proton pump inhibitor‐induced hypomagnesemia

**DOI:** 10.1111/apha.13846

**Published:** 2022-06-14

**Authors:** Lisanne M. M. Gommers, Joost G. J. Hoenderop, Jeroen H. F. de Baaij

**Affiliations:** ^1^ Department of Physiology, Radboud Institute for Molecular Life Sciences Radboud University Medical Center Nijmegen the Netherlands

**Keywords:** gut microbiome, magnesium, omeprazole, PPI, proton pump inhibitor

## Abstract

Proton pump inhibitors (PPIs) reliably suppress gastric acid secretion and are therefore the first‐line treatment for gastric acid‐related disorders. Hypomagnesemia (serum magnesium [Mg^2+^] <0.7 mmol/L) is a commonly reported side effect of PPIs. Clinical reports demonstrate that urinary Mg^2+^ excretion is low in PPI users with hypomagnesemia, suggesting a compensatory mechanism by the kidney for malabsorption of Mg^2+^ in the intestines. However, the exact mechanism by which PPIs cause impaired Mg^2+^ absorption is still unknown. In this review, we show that current experimental evidence points toward reduced Mg^2+^ solubility in the intestinal lumen. Moreover, the absorption pathways in both the small intestine and the colon may be reduced by changes in the expression and activity of key transporter proteins. Additionally, the gut microbiome may contribute to the development of PPI‐induced hypomagnesemia, as PPI use affects the composition of the gut microbiome. In this review, we argue that the increase of the luminal pH during PPI treatment may contribute to several of these mechanisms. Considering the fact that bacterial fermentation of dietary fibers results in luminal acidification, we propose that targeting the gut microbiome using dietary intervention might be a promising treatment strategy to restore hypomagnesemia in PPI users.

## 1 INTRODUCTION

Proton pump inhibitors (PPIs) are the first‐line treatment for gastric acid‐related disorders such as peptic ulcer disease (PUD), gastroesophageal reflux disease (GERD), and non‐steroid anti‐inflammatory drug (NSAID)‐induced mucosal damage.^1^ In the United States alone, PPIs are used by 14.9 million patients receiving almost 160 million prescriptions annually.^2^ PPI use is underestimated because PPIs are also available by over‐the‐counter sale. In general, PPIs have an excellent safety profile.^3^, ^4^ However, there is increasing concern in recent years about high prescription numbers and prolonged usage of PPIs.^3^, ^5^, ^6^, ^7^

In the last decade, PPI intake has been associated with an increasing number of serious side effects, including increased risk of infections, micronutrient deficiencies, fractures, diabetes mellitus, and kidney and cardiovascular disease.^8^, ^9^, ^10^, ^11^, ^12^ PPI use has been demonstrated to cause Mg^2+^ deficiency (hypomagnesemia).^13^, ^14^, ^15^, ^16^, ^17^ PPI‐induced hypomagnesemia is associated with clinical complaints including fatigue, muscle cramps, and arrhythmias.^18^, ^19^ In general, PPI‐induced hypomagnesemia occurs during long‐term PPI treatment (>1 year). Upon PPI withdrawal, serum Mg^2+^ levels rapidly restore within several days to the normal concentration range ([Mg^2+^] 0.7–1.0 mmol/L) but decrease again after re‐challenge with PPIs.^20^ These effects are independent of the type of PPI.^21^

In this review, an overview will be provided of the clinical studies that describe the prevalence and risk factors for the development of hypomagnesemia during PPI therapy. Moreover, we aim to describe the molecular mechanisms underlying the disease, as significant progress has been made toward our understanding of PPI‐induced hypomagnesemia. Based on recent advances, we will propose novel therapeutic approaches toward the treatment of PPI‐induced hypomagnesemia.

## 2 PROTON PUMP INHIBITORS

PPIs prevent gastric acid secretion by direct inhibition of the gastric proton‐potassium ATPase (H^+^, K^+^‐ATPase) of the epithelial cell lining in the mucosa of the stomach.^22^ Orally administered PPIs are taken up in the small intestine and released to the circulation. Consequently, PPIs accumulate in the acidic secretory canaliculus of the parietal cell. The acidic environment allows the conversion of PPI‐prodrugs into active metabolites that block the gastric H^+^, K^+^‐ATPase. This mechanism contributes to the specificity of PPIs for the gastric H^+^, K^+^‐ATPase and reduces inhibition of non‐gastric H^+^, K^+^‐ATPases.^23^ Clinical studies have shown that PPIs reliably suppress acid secretion up to 24 h (pH >4).^22^ PPIs are more potent inhibitors of gastric acid secretion than alternative drugs, including histamine‐2‐receptor‐blockers (H_2_RAs) or anticholinergics.^22^, ^23^ Consequently, patients are often dependent on the use of PPIs since they do not respond sufficiently to H_2_RAs.^24^

### 2.1 PPI ‐induced hypomagnesemia

PPI‐induced hypomagnesemia was first reported in 2006.^25^ Since then, numerous clinical studies have confirmed that PPI use leads to hypomagnesemia.^11^, ^26^, ^27^, ^28^, ^29^, ^30^, ^31^, ^32^, ^33^ As PPI users with mild hypomagnesemia (± 0.6 mmol/L) are often asymptomatic, PPI‐induced hypomagnesemia is easily missed because routine serum Mg^2+^ measurements during PPI therapy are often not performed.^11^ By systematic analysis of cohort studies on the prevalence of PPI‐induced hypomagnesemia, we demonstrate that hypomagnesemia is a common side effect of PPI therapy (Table 1). The reported prevalence is approximately 19% (range: 2%–36%).^14^ Indeed, PPI treatment increases the risk for the development of hypomagnesemia with an odds ratio (OR) of 1.83 (individual studies report ORs between 1.0 and 5.4, Table 1).^14^

**TABLE 1:** The association between PPI use and the development of hypomagnesemia^a^

Author	Study design	Population	Cut‐off value hypomagnesemia (mmol/L)	No. of PPI users	No. of non‐users	Cases of PPI‐induced hypomagnesemia (%)	Risk assessment^b^ (95% CI)	Adjustment variables
Danziger et al.^26^ 2013	Cross‐sectional	Inpatients	<0.80	2632	8858	405 (15.3%)	OR: 1.10 (0.96–1.25)	Age, sex, ethnicity, comorbidities, diuretics, renal function, systolic blood pressure, heart rate, temperature, serum calcium, serum phosphorus, serum glucose, hematocrit
Douwes et al.^27^ 2019	Cross‐sectional	Inpatients	<0.70	389	300	102 (26.0%)	OR: 2.00 (1.21–3.31)	Age, sex, BMI, eGFR, proteinuria, time since kidney transplantation, alcohol, diabetes mellitus, cardiovascular disease, diuretics, tacrolimus, cyclosporine, immunosuppressants, dietary magnesium intake
Gau et al.^28^ 2012	Cross‐sectional	Inpatients	<0.70	207	280	48 (2.3%)	OR: 2.50 (1.43–4.36)	Age, sex, diabetes mellitus, heart failure, diuretics, magnesium, and potassium supplementation, acute GI illness, serum albumin, serum potassium, serum creatinine
Kieboom et al.^29^ 2015	Cross‐sectional	Outpatients	<0.80	724	9094	36 (5.0%)	OR: 2.00 (1.36–2.93)	Age, sex, BMI, eGFR, diabetes mellitus, stroke, coronary heart disease, hypertension, alcohol use, diuretics
Kim et al.^11^ 2015	Case–control	Inpatients	<0.70	105	210	32 (35.8%)	OR: 5.39 (1.06–27.49)	Age, sex, comorbidities, drugs, electrolyte levels (sodium, potassium, calcium, urea, creatinine, albumin)
Lindner et al .^30^ 2014	Cross‐sectional	Inpatients	<0.75	423	4695	155 (3.6%)	OR: 2.10 (1.54–2.85)	CCL score, eGFR
Markovits et al.^31^ 2014	Cross‐sectional	Outpatients	<0.70	22 458	69 714	2532 (11.0%)	OR: 1.66 (1.55–1.78)	Age, sex, diabetes mellitus, hypertension, heart failure, eGFR, diuretics, immunosuppressants, lithium, dioxin, recent hospitalization
Pasina et al.^32^ 2015	Cross‐sectional	Inpatients	<0.80	299	305	63 (21.0%)	OR: 4.31 (2.49–7.86)	Age, sex, diabetes mellitus, chronic diarrhea, malabsorption, alcohol
Sutton et al.^33^ 2019	Cross‐sectional	Inpatients	<0.80	329	5718	31 (9.0%)	HR: 3.16 (2.56–3.90)	Age, sex, ethnicity, CCL score, alcohol, viral suppression, index year

Abbreviations: BMI, body mass index; CCL score, Charlson Comorbidity Index score; CI, confidence interval; eGFR, estimated glomerular filtration rate; GI, gastrointestinal; HR, hazard ratio; OR, odds ratio.

^a^ Articles were obtained after PubMed search using the following search terms in April 2020: “proton pump inhibitor” OR “omeprazole” OR “esomeprazole” OR “lansoprazole” OR “dexlansoprazole” OR “pantoprazole” OR “rabeprazole” AND “hypomagnesemia”. Articles were only included if the study reported on the type of PPI, specified the cut‐off value for hypomagnesemia, patient population, number of PPI users, data on serum Mg^2+^ status, confounding factors, and included a risk assessment on PPI use and hypomagnesemia.

^b^ Risk assessment describes the risk to develop hypomagnesemia during PPI therapy.

Heterogeneity in design, population, hypomagnesemia cut‐off value, and adjustment variables may explain the variation in the prevalence of hypomagnesemia among studies. A recent meta‐analysis showed that the incidence of PPI‐induced hypomagnesemia is similar in outpatients and hospitalized patients.^15^ Similarly, there were no changes in the incidence of hypomagnesemia with different cut‐off values.^15^ Other factors may therefore explain the variability in the development of PPI‐induced hypomagnesemia between populations. We and others demonstrated that diuretics‐use and genetic variants (SNPs) in Mg^2+^ channel TRPM6 increase the risk for PPI‐induced hypomagnesemia.^29^, ^34^, ^35^, ^36^ A meta‐analysis of 12 studies showed that the PPI dose is associated with the development of hypomagnesemia (high dose OR 2.13; 95% CI 1.26–3.59).^14^ Additionally, the prolonged duration of PPI treatment may be an additional factor. A PPI use of more than 6 months was associated with a higher risk (OR 2.99; 95% CI 1.73–5.15) to develop hypomagnesemia.^29^ Altogether, the treatment duration and PPI dosage are demonstrated to be important factors for the development of PPI‐induced hypomagnesemia.

Urinary Mg^2+^ excretion is generally reduced in patients with PPI‐induced hypomagnesemia.^25^, ^37^, ^38^, ^39^, ^40^, ^41^, ^42^ This observation suggests that the kidney is compensating for reduced intestinal Mg^2+^ absorption, excluding renal loss as cause for Mg^2+^ deficiency. Importantly, PPI use did not affect dietary Mg^2+^ intake or renal function that could cause the urinary Mg^2+^ loss. Therefore, it is postulated that PPI‐induced hypomagnesemia is caused by impaired intestinal Mg^2+^ absorption.

### 2.2 Intestinal Mg^2+^ absorption

Approximately 30%–50% of the daily Mg^2+^ intake is absorbed in the gastrointestinal (GI) tract (± 100 mg, resulting a recommended daily intake of 300–350 mg).^17^ However, the absorption rate may be higher (up to 80%), when the dietary Mg^2+^ intake is low.^43^

**FIGURE 1:**
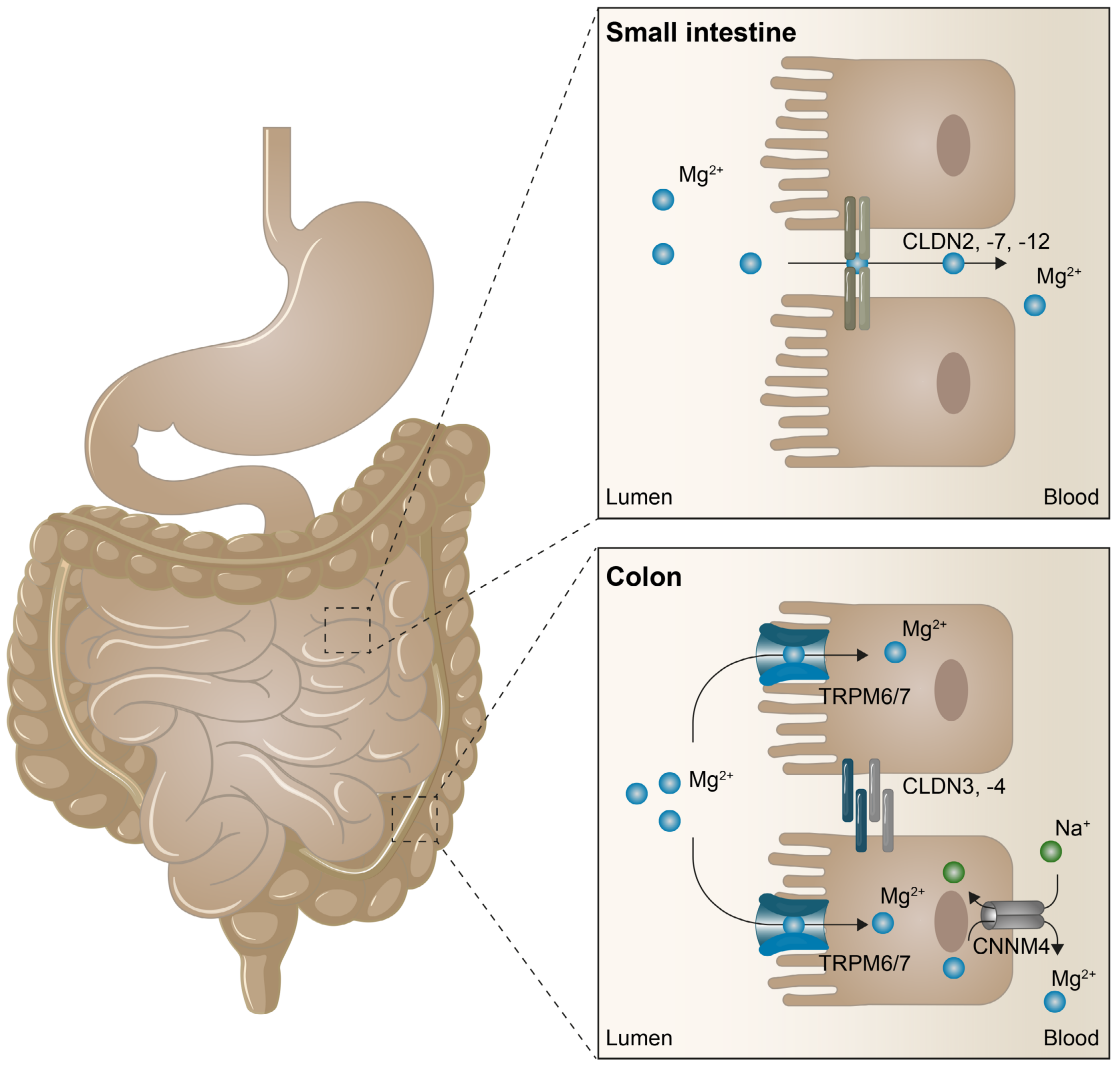
Intestinal Mg^2+^ absorption pathways Mg^2+^ absorption is mediated by two separate absorption pathways. In the small intestine, Mg^2+^ absorption is mainly of paracellular nature through tight junction complexes between adjacent epithelial cells. Here, CLDN2, −7, and − 12 enhance paracellular permeability. In the large intestine, Mg^2+^ is absorbed via active, transcellular transport facilitated by TRPM6/7 channels. Extrusion of Mg^2+^ to the blood compartment is mediated by CNNM4 on the basolateral side of the colonocytes.

Two independent absorption pathways facilitate intestinal Mg^2+^ absorption (Figure 1). First, passive transport via tight junction complexes of two neighboring epithelial cells allows mass Mg^2+^ absorption.^44^ This paracellular route consists of occludins, claudins, and E‐cadherin, that maintain the intestinal barrier integrity and facilitate the transport of ions, nutrients, and water.^45^ Claudin‐1, −3, −4, −5, and − 8 are known for their tightening properties of intestinal epithelium.^46^ They show high expression levels in the colon but are hardly expressed in the small intestine, making this segment very permeable to ions.^47^ Claudin‐2, −7, and − 12 are selective for cations and enhance the paracellular permeability in duodenum and ileum.^47^ In particular, the lumen‐negative transepithelial electrical potential (± 5 mV) across the tight junction determines the permeability.^48^ In the small intestine, Mg^2+^ absorption is facilitated mainly via the paracellular absorption route. Indeed, luminal Mg^2+^ concentrations, and Mg^2+^ absorption rates are linearly correlated.^49^

In the colon and distal segments of the ileum, fine‐tuning of Mg^2+^ absorption is mediated via channels of the transient receptor potential melastatin (TRPM) family. This absorption pathway is transcellular and secondary active. Transcellular transport of Mg^2+^ accounts for ~30% of total Mg^2+^ absorption in normal physiological conditions.^17^ TRPM6 and TRPM7 channels are expressed on the luminal side and cyclin M4 (CNMM4) Na^+^‐Mg^2+^ exchangers on the basolateral side of the intestinal epithelial cell.^50^ TRPM6/7 channels form heterotetramers and facilitate Mg^2+^ uptake.^51^ TRPM6 reduces the inhibition of TRPM7 by Mg‐ATP sensitivity and thereby increases the permeability for Mg^2+^_._ ^52^ Mutations in the TRPM6 gene are causative for hypomagnesemia with secondary hypocalcemia (HSH), an autosomal recessive genetic disorder characterized by extremely low serum Mg^2+^ levels (0.1–0.3 mmol/L).^17^ This hereditary disease highlights the importance of TRPM6 for intestinal Mg^2+^ absorption. Moreover, it was shown that TRPM6 in the intestine, but not in the kidney, is essential to maintain systemic Mg^2+^ balance in mice.^52^ Indeed, intestine‐specific disruption of *Trpm6* in mice caused severe hypomagnesemia due to a defect in intestinal Mg^2+^ absorption.^52^

## 3 PPI ‐INDUCED HYPOMAGNESEMIA IS CAUSED BY INTESTINAL MALABSORPTION OF MG^2+^

Over the last years, several experimental studies have addressed the putative molecular mechanisms by which PPIs affect Mg^2+^ absorption along the intestinal tract. Here, we will critically describe the evidence for these mechanisms as potential underlying cause for the development of PPI‐induced hypomagnesemia.

### 3.1 PPIs affect paracellular transport of Mg^2+^ in the small intestine

Mg^2+^ absorption in the small intestine depends on passive paracellular diffusion. Consequently, two factors are essential to consider: Mg^2+^ availability and tight junction permeability. Both factors are potentially compromised by PPI treatment.

#### 3.1.1 Luminal pH of the small intestine

**FIGURE 2:**
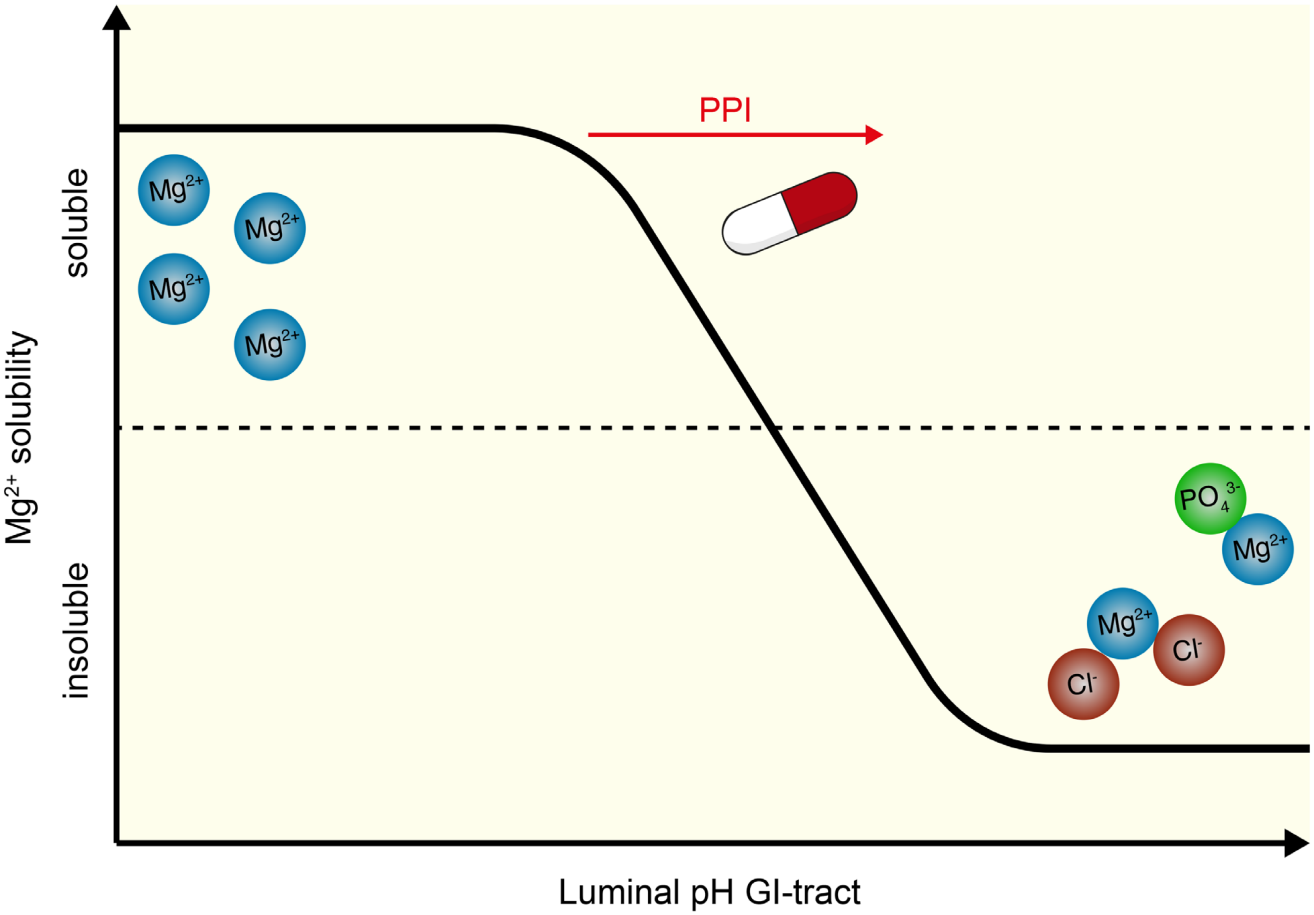
Hypothesis of the effects of PPIs on the Mg^2+^ solubility in the gastrointestinal tract. Schematic representation in which PPIs increase the luminal pH of the gastrointestinal (GI) tract and thereby affect Mg^2+^ solubility. At higher luminal pH, Mg^2+^ binds negatively charged molecules, such as Cl^−^ and PO_4_^3−^, resulting in reduced Mg^2+^ availability for absorption.

PPIs have a direct effect on the luminal pH of the small intestine. In patients with pancreatitis, the gastric pH was correlated with the small intestinal pH during PPI treatment.^53^ In general, an increased gastric pH by 2 pH units translates into a 1 pH unit increase in the small intestine.^53^ Moreover, the luminal pH of the stomach, duodenum, and jejunum, but not of cecum and colon, are increased by a single omeprazole dose in Sprague–Dawley rats.^54^ Consequently, paracellular Mg^2+^ transport in duodenum, jejunum, and ileum was reduced by 81%, 71%, and 69%, respectively.^54^ Similarly, long‐term omeprazole treatment in Sprague–Dawley rats showed reduced duodenal Mg^2+^ absorption as result of a higher luminal pH.^55^ The diminished Mg^2+^ absorption can be explained by the decreased solubility of Mg^2+^ at a higher pH.^56^ Moreover, Mg^2+^ in the GI tract is partially bound to proteins and negatively charged ions such as Cl^−^ and PO_4_^3−^. This is also the reason why Mg^2+^‐salts are effective oral phosphate binders and are used for the treatment of chronic kidney disease (CKD).^57^ However, the effectiveness is dependent on the luminal pH as Mg^2+^ salts bind more phosphate in an alkaline environment.^58^ Together, PPIs increase the luminal pH of the segments of the small intestine and thereby might reduce the Mg^2+^ solubility and consequently absorption (Figure 2).

#### 3.1.2 Modulation of paracellular permeability

**FIGURE 3:**
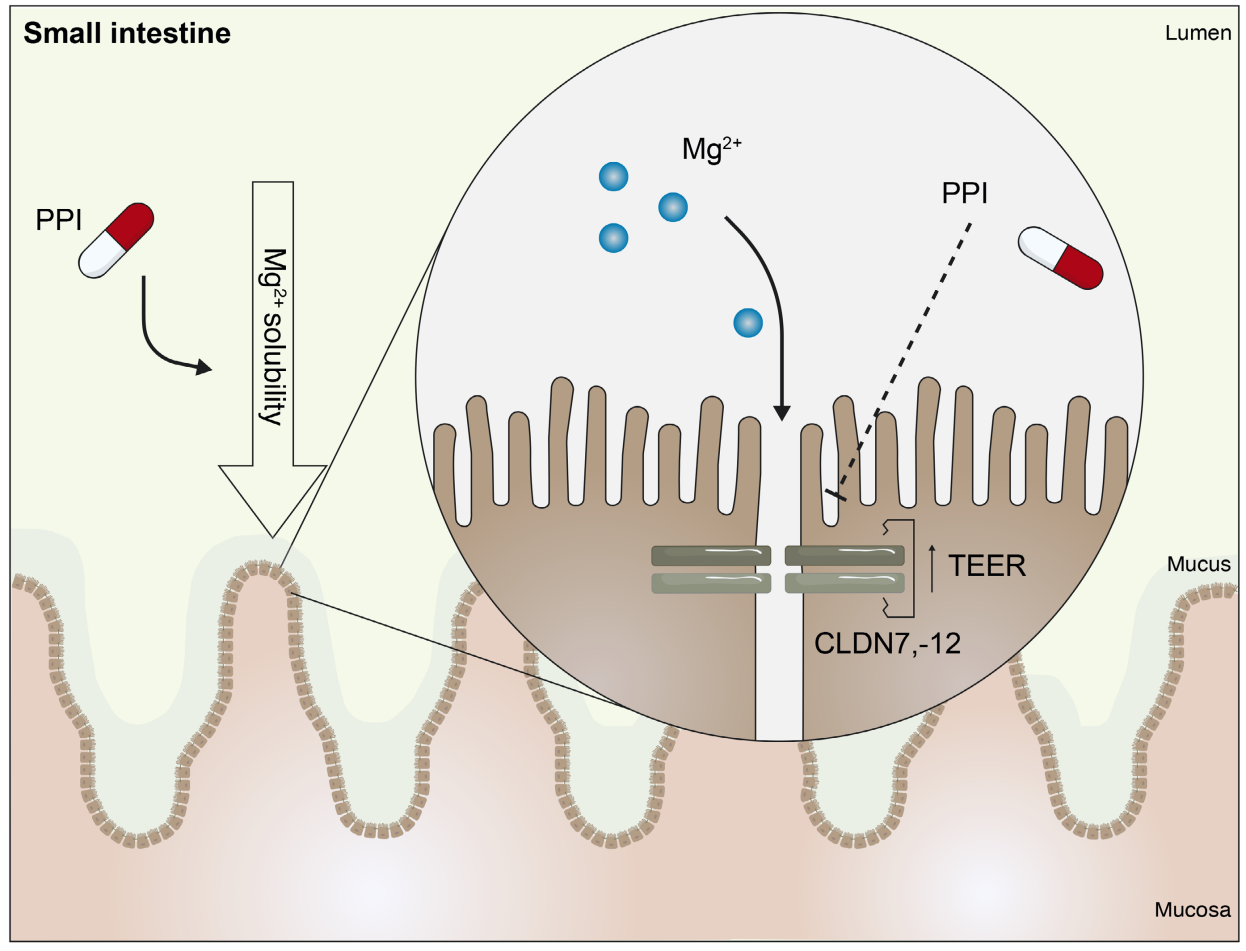
PPIs impair Mg^2+^ absorption in the small intestine During PPI therapy, the luminal pH of the small intestine increases. Consequently, Mg^2+^ solubility and absorption are reduced. Moreover, PPIs lower the expression of CLDN7, −12 and increase the transepithelial electrical resistance (TEER). Consequently, Mg^2+^ absorption in the small intestine is decreased.

PPIs affect paracellular permeability by direct and indirect mechanisms. Caco‐2 cells treated with omeprazole (200–600 ng/mL) for 14 and 21 days showed reduced paracellular transport as measured by Mg^2+^ fluxes over Caco‐2 cell monolayers.^59^ Omeprazole‐treated Caco‐2 cells demonstrate reduced protein expression of permeability‐enhancing claudins, including claudin‐7 and ‐12, but not claudin‐2.^60^ Consequently, the transepithelial electrical resistance (TEER) was increased, suggesting that omeprazole reduced the paracellular permeability^59^ (Figure 3). Moreover, PPIs also indirectly influence paracellular permeability by increasing the luminal pH. Lowering the apical pH from 7.4 to 5.5 increased the protein expression of CLDN‐7 and ‐12 in Caco‐2 cells.^60^ Under these conditions, the inhibitory effect of omeprazole was abolished and paracellular transport of Mg^2+^ was enhanced.^60^ This study suggests that luminal pH and intestinal permeability are closely linked.

### 3.2 PPIs affect transcellular transport of Mg^2+^ in the colon

The colon has been the main focus of studies toward the mechanisms of PPI‐induced hypomagnesemia. In this segment, a significant amount of Mg^2+^ transport is absorbed in healthy subjects. Moreover, absorption in the colon can compensate for reduced Mg^2+^ absorption in the small intestine. Although the colon is spatially separated from the stomach where PPIs predominantly act, a growing body of evidence demonstrates that colon is also affected by PPI therapy.

#### 3.2.1 Luminal pH of the colon

The colonic H^+^, K^+^‐ATPase (*ATP12A*) is a close homolog of the gastric H^+^, K^+^‐ATPase (*ATP4A*).^61^ Consequently, it has been hypothesized that omeprazole can inhibit colonic H^+^, K^+^‐ATPases, resulting in a less acidic local pH. Indeed, omeprazole treatment significantly increased the mRNA expression of colonic H^+^, K^+^‐ATPases in PPI‐treated mice.^62^ An increased intraluminal pH in the colon would directly affect the solubility of Mg^2+^, as discussed in previous sections. Moreover, pH may also directly affect the activity of the Mg^2+^ channels TRPM6 and TRPM7 in the colon. However, whether PPIs have direct effects on the colonic H^+^, K^+^‐ATPase is still heavily debatable, as PPIs will not accumulate in these cells, in contrast to the parietal cells of the stomach.

#### 3.2.2 TRPM6 function

**FIGURE 4:**
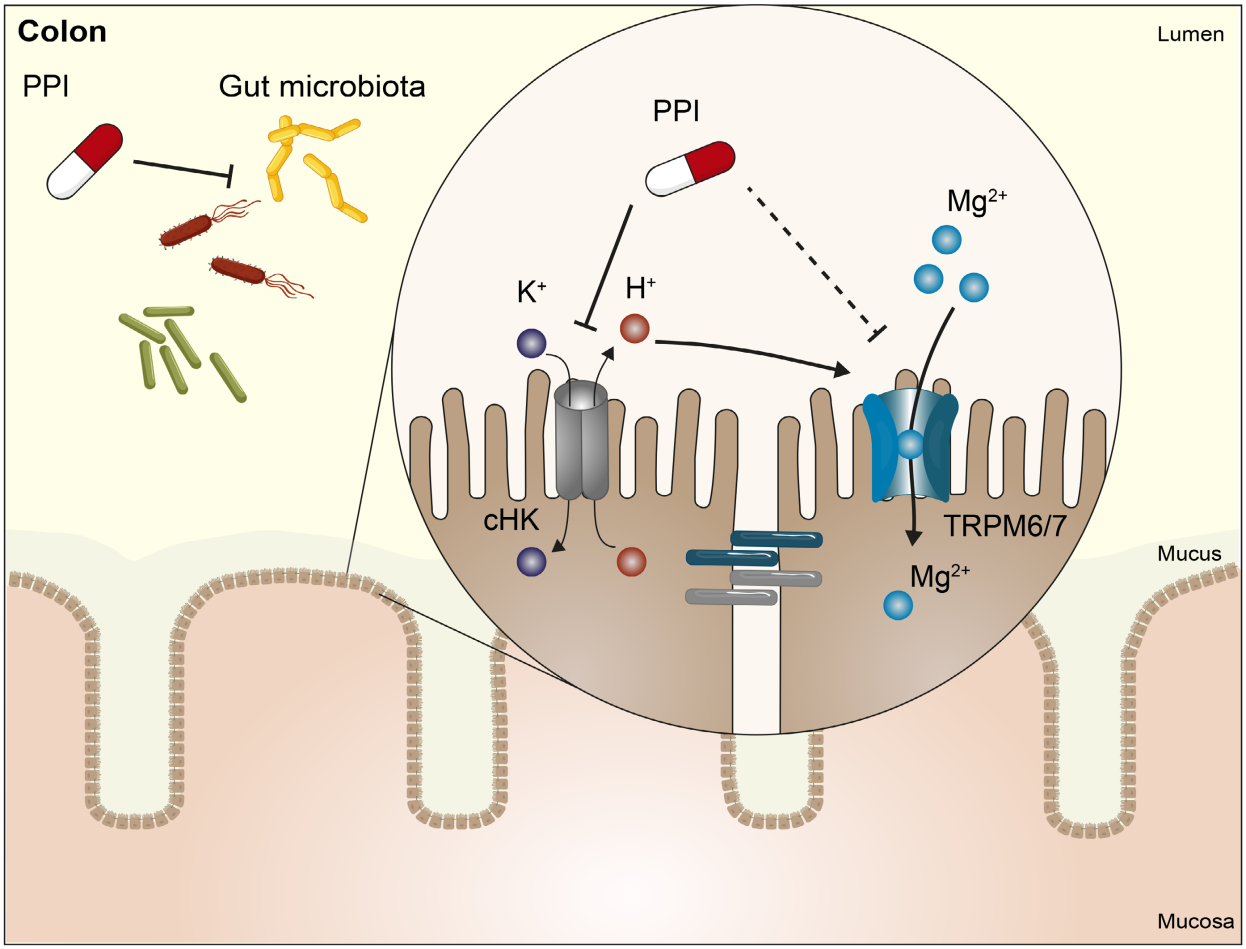
PPIs affect Mg^2+^ absorption in the colon. PPIs affect the composition and diversity of the gut microbiome. Additionally, PPIs inhibit the colonic H^+^, K^+^‐ATPases (cHK, *ATP12A*) making the pH of the colon less acidic. These factors might reduce the activity of TRPM6 channels.

The pH in the colon may also determine the activity TRPM6/7, which represents the luminal Mg^2+^ channel in the colon.^63^ Given that TRPM6 activity is higher at lower pH,^63^ the omeprazole‐induced increase in colonic pH might reduce TRPM6‐mediated Mg^2+^ absorption (Figure 4). Omeprazole treatment was shown to diminish colonic Mg^2+^ absorption by 39% in Sprague–Dawley rats.^54^ Despite an increased protein expression of TRPM6 in the colon of omeprazole‐treated rats, which may be a compensatory response for reduced absorption.^54^ Increased colonic *Trpm6* expression has been demonstrated previously in omeprazole‐treated mice.^62^ Genetic studies also confirm the essential role of TRPM6 in PPI‐induced hypomagnesemia. People with two single nucleotide polymorphisms (SNPs) in the *TRPM6* gene (rs3750425 and rs2274924) have an increased risk for the development of hypomagnesemia in response to PPI treatment.^36^ Nevertheless, the mechanisms by which PPIs affect TRPM6 are largely unresolved.

#### 3.2.3 Gut microbiome

Importantly, bacterial fermentation results in acidification of the colon. This has been shown beneficial for the solubility and absorption of Mg^2+ 43^. Therefore, it is interesting that PPIs have consistently been shown to change the composition of the gut microbiome (Table 2).^64^, ^65^, ^66^, ^67^, ^68^, ^69^, ^70^, ^71^, ^72^, ^73^ In particular, PPI users have generally a lower gut microbial diversity^64^, ^66^, ^67^, ^69^, ^70^, ^71^, ^72^, ^73^ (Figure 4). The bacterial richness and evenness (alpha diversity) of the gut microbiota is lower in PPI users compared to non‐users.^66^, ^67^ Additionally, the majority of studies also report significant differences in overall bacterial composition (beta diversity).^64^, ^66^, ^67^, ^72^, ^73^ All studies reporting microbial changes in response to PPI treatment have been summarized in Table 2. In general, differences in overall bacterial composition are more common than differences in species richness. At the taxonomic level, PPI use is associated with an increase in the abundance of Firmicutes, including bacteria from the order Lactobacillales (with families Enterococcaceae, Lactobacillaceae, and Streptococcaceae).^64^, ^66^, ^68^, ^69^, ^70^, ^72^, ^73^ The PPI‐induced disturbances in the gut microbiota may be the result of changes in the luminal pH. It is hypothesized that more (pathogenic) bacteria will survive the stomach with subsequent changes in the gut microbiome.^74^ Indeed, several studies have shown that species in the oral microbiota are significantly enriched in the fecal microbiota of PPI users.^66^, ^67^ Loss of this barrier might increase the risk for enteric infections. Indeed, PPI use has been previously shown to increase the risk for the development of *Clostridium difficile* (CDI) infection (OR 1.26; 95% CI 1.12–1.39) or small intestinal bacterial overgrowth (SIBO) (OR 2.28; 95% CI 1.24–4.21).^75^, ^76^

**TABLE 2:** PPI use is associated with changes in the gut microbiome^a^

Author^a^	Study design (population)	PPI users vs non‐users	Duration of PPI treatment	Sample	Sequencing technique	Changes at the taxonomic level^b^	Changes in diversity	
							Alpha (metric)	Beta (metric)
Clooney et al.^64^ 2016	Cross‐sectional cohort study from population‐based database (Canada)	32 vs. 29	>5 years	Feces	16S	↑ f_Lachnospiraceae ↑ f_Streptococcaceae	No difference (Shannon index) (Chao1 richness)	Significant shift (Bray‐Curtis) (UniFrac PCA)
Freedberg et al.^65^ 2015	Prospective open‐label trial (healthy adults)	12	4–8 weeks	Feces	16S	↑ f_Streptococcaceae ↑ f_Enterococcaceae	No difference (Shannon index)	No difference (weighted UniFrac PCA)
Imhann et al.^66^ 2016	Cross‐sectional cohort study from population‐based cohorts (Lifelines‐DEEP, IBN UMCG, IBS MUMC)	211 vs. 1604	Not reported	Feces	16S	↑ f_Lactobacillaceae ↑ f_Enterococcaceae ↑ f_Streptococcaceae ↑ f_Micrococcaceae ↓ f_Bifidobacteriacaea	Decreased (Shannon index) (Chao1 richness )	Significant shift (PCoA)
Jackson et al.^67^ 2016	Cross‐sectional cohort study (healthy twins: TwinsUK)	229 vs. 1598	>3 years	Feces	16S	↑ f_Streptococcaceae ↑ f_Lactobacillaceae ↑ f_Micrococcaceae ↓ f_Ruminococcaceae	Decreased (Shannon index) (OTU counts) (Chao1 richness)	Not determined
Mishiro et al.^68^ 2018	Prospective open label trial (healthy adults)	10	4 weeks	Feces	16S	↑ g_*Streptococcus*	No difference (Shannon index) (Chao1 richness)	No difference (Bray‐Curtis) (unweighted UniFrac)
Otsuka et al.^69^ 2017	Prospective open label trial (healthy adults)	11	4 weeks	Feces	16S	↑ g_*Streptococcus* ↑ g_*Bacteroides*	No difference (Shannon index) (Chao1 richness)	Significant shift (UniFrac PCA)
Reveles et al.^70^ 2018	Prospective open‐label trial (healthy elderly adults)	24	2 weeks	Feces	16S	↑ f_Streptococcaceae ↓ f_Lachnospiraceae ↓ f_Bifidobacteriacaea	No difference (Shannon index)	Significant shift (Bray‐Curtis)
Seto et al.^71^ 2014	Prospective open‐label trial (healthy adults)	9	4 weeks	Feces	16S	No differences	Decreased (OTU counts) (Chao1 richness)	No difference (unweighted UniFrac)
Takagi et al.^72^ 2018	Cross‐sectional cohort study (outpatients)	36 vs. 36	>1 year	Feces	16S	↑ g_*Streptococcus* ↑ g_*Ruminococcus* ↓ g_*Faecalibacterium*	No difference (Shannon index) (Chao1 richness)	Significant shift (UniFrac PCA)
Tsuda et al.^73^ 2015	Cross‐sectional cohort study (outpatients)	18 vs. 27	>2 year	Feces	16S	↑ g_*Streptococcus* ↓ g_*Faecalibacterium*	No difference (Shannon index)	Significant shift (UniFrac PCA)

Abbreviations: 16S, 16S rRNA sequencing; OTU, operational taxonomic unit; PCA, principal component analysis; PCoA, principal coordinates analysis; UniFrac, unique fraction.

^a^ Articles were obtained after PubMed search using the following search terms in April 2020: “proton pump inhibitor” AND “microbiome” OR “microbiota”.

^b^ Taxonomic composition was described as k_, kingdom; p_, phylum; c_, class; o_, order; f_, family; g_, genus; s_, species. ↑↓ describe the direction of change.

#### 3.2.4 Short‐chain fatty acids

Bacterial fermentation has been associated with increased production of important end‐metabolites, including short‐chain fatty acids (SCFAs; acetate, propionate, butyrate). However, in a recent animal study, omeprazole did not affect the concentrations of colonic SCFAs in mice with hypomagnesemia, despite the profound effects of PPIs on the gut microbiome composition.^77^ In twelve patients with reflux esophagitis, the concentrations of SCFAs were also not altered after 8 weeks of PPI treatment.^78^ This finding suggests that the malabsorption of Mg^2+^ is not caused by direct effects of SCFAs, but likely by changes in the luminal environment of the colon. Targeting the gut microbiota with prebiotic inulin fibers (20 g/day) improved serum Mg^2+^ levels in patients with PPI‐induced hypomagnesemia.^79^ Similar results were reported by Coudray et al. showing that inulin lowered the cecal pH and increased Mg^2+^ solubility from 13% to 75%–95% compared to fructose treatment. These changes significantly increased the Mg^2+^ absorption compared to a fiber‐free diet in rats.^80^ Altogether, these studies further highlight the importance of an acidic luminal environment for the absorption of Mg^2+^ in the colon.

### 3.3 Treatment of PPI ‐induced hypomagnesemia

Currently, there are no adequate treatment strategies to restore hypomagnesemia in PPI users. PPI withdrawal still remains the gold standard.^20^ Moreover, oral Mg^2+^ supplementation is often insufficient and causes diarrhea, nausea, and abdominal cramping at high concentrations.^17^ In this section, we discuss the aforementioned treatment options, as well as future research strategies aiming to acidify the intestinal lumen in PPI users with hypomagnesemia.

#### 3.3.1 Withdrawal

Clinical case reports show that serum Mg^2+^ levels restore to physiological concentrations upon PPI withdrawal,^20^ but reappear after re‐challenge with a PPI. Both observations occur within days to weeks and were independent of type of PPI.^20^ However, discontinuation of PPI therapy might not always be possible and, therefore, switching to different acid suppressants, such as H_2_RAs, could be considered. However, it is still debatable whether H_2_RAs also cause hypomagnesemia. Kieboom et al. demonstrated a positive correlation between H_2_RA use and hypomagnesemia (OR: 2.19; 95% CI 1.21–3.98),^29^ while this was not observed in a different patient cohort (OR 1.06; 95% CI 0.54–2.06).^35^ In previous sections, we have pointed out that treatment duration and daily dose are important contributing factors for the development of PPI‐induced hypomagnesemia. Therefore, long‐term use and high doses of PPI treatment should be prevented by healthcare professionals.

#### 3.3.2 Mg^2+^ supplementation

Oral Mg^2+^ supplementation does not fully restore serum Mg^2+^ levels in patients with PPI‐induced hypomagnesemia. Indeed, high dose (30–40 mmol/day) oral Mg^2+^ supplements only partly restored serum Mg^2+^ levels in two PPI users with severe hypomagnesemia.^37^ Only PPI withdrawal completely resolved the hypomagnesemia in these patients.^37^ Additionally, intravenous Mg^2+^ infusions did not correct the Mg^2+^ deficiency in PPI users as shown by the consistently low levels of Mg^2+^ in the urine.^41^ In the same study, oral Mg^2+^ supplementation only shortly maintained serum Mg^2+^ levels within the normal range.^41^ Considering the fact that high oral Mg^2+^ supplementation often causes diarrhea, nausea, and abdominal cramping, this treatment option is both ineffective and poorly tolerated.^17^

#### 3.3.3 Prebiotics

PPIs greatly affect the luminal pH of the GI tract. Dietary fibers might therefore be a promising treatment strategy to target the microbiome and acidify the lumen of the colon. Recent intervention studies demonstrate that pre‐ and probiotic approaches can reduce PPI‐induced side effects, such as enteric infections and mineral deficiencies. Supplementation with *Lactobacillus reuteri* reduced the prevalence of enteric infections after 12 weeks of treatment in children with GERD.^81^ Similar results were observed in reflux esophagitis patients that were treated for 8 weeks with a probiotic cocktail of *Bacillus subtilis* and *Enterococcus faecium*.^82^ A recent meta‐analysis demonstrated that probiotics improve GERD‐related symptoms, including the frequency and duration of reflux episodes.^83^

Moreover, prebiotic fibers have been previously shown to improve mineral absorption. Inulin supplementation successfully increased Ca^2+^ and Mg^2+^ in postmenopausal women.^84^ Dietary intake of fructose oligosaccharides improved Mg^2+^ absorption by 18% after 36 days in adolescent girls using a stable isotope technique.^85^ A recent study in PPI users with hypomagnesemia demonstrated that inulin fibers for 14 days significantly increased serum Mg^2+^ levels.^79^ Importantly, this microbiome‐targeting therapy with prebiotics improved hypomagnesemia‐related symptoms, including generalized weakness, tetany of hands, and muscle cramps.^79^

## 4 PPI ‐INDUCED HYPOMAGNESEMIA: DIGESTING CURRENT HYPOTHESES

Hypomagnesemia is a well‐known side effect of PPIs. Treatment duration (>1 year) and daily dose are important contributing factors for the development of PPI‐induced hypomagnesemia. Current clinical and experimental studies point to malabsorption of Mg^2+^ in the GI tract as underlying cause for the development of hypomagnesemia. This observation sets PPI‐induced hypomagnesemia apart from all other forms of drug‐induced hypomagnesemia that are characterized by renal Mg^2+^ wasting, as seen in users of gentamycin, calcineurin inhibitors, diuretics, and anti‐diabetic drugs.^86^

In this review, we set out numerous mechanisms by which PPIs affect Mg^2+^ absorption in both small intestine and colon. Although the exact molecular mechanism remains to be elucidated, most evidence points toward reduced Mg^2+^ solubility in the small intestine or changes in the composition and function of the gut microbiome in the colon. This is likely caused by the increase of the luminal pH during PPI treatment. Future studies using wireless pH monitoring capsules are required to better understand the physiological pH range of different intestinal segments as well as the direct effects of PPIs on the luminal pH. This would allow to determine the optimal pH range for Mg^2+^ solubility. Considering the fact that high oral Mg^2+^ supplementation does not recover serum Mg^2+^ levels in PPI users with hypomagnesemia it is very likely that PPIs mainly impair active Mg^2+^ absorption in the colon rather than passive absorption in the small intestine.

To date, the cornerstone of hypomagnesemia treatment is still PPI withdrawal. However, this is not possible for patients who are dependent on PPIs. Alternative treatment options, such as oral Mg^2+^ supplementation or the use of different acid suppressants, are less effective. The gut microbiome might be a novel target to ameliorate PPI‐induced side effects using prebiotic strategies. This might rely on common mechanisms: (i) diets rich in fibers have been associated with increased bacterial diversity^87^, ^88^; (ii) fermentation of dietary fibers increases the production of SCFAs and acidification of the intraluminal pH.^89^, ^90^ Clinical trials studies are required to examine which dietary fibers optimally enhance intestinal Mg^2+^ absorption in PPI users with hypomagnesemia.

## CONFLICT OF INTEREST

No conflicts of interest.
